# Editorial: Application of radiomics in understanding tumor biological behaviors and treatment response

**DOI:** 10.3389/fonc.2023.1257447

**Published:** 2023-08-18

**Authors:** Ningping Xiao, Zhengda Pei, Wenhui Lu, Rongyao Fang, Yi Jin, Guanzhi Zhou, Xue Meng, Sweet Ping Ng, Lei Xing, Zhongxing Liao, Nanna Maria Sijtsema, Pei Yang

**Affiliations:** ^1^ Hunan Cancer Hospital, Xiangya School of Medicine, Central South University, Changsha, Hunan, China; ^2^ Graduate Collaborative Training Base of Hunan Cancer Hospital, Hengyang Medical School, University of South China, Hengyang, Hunan, China; ^3^ Department of Radiotherapy, University Medical Center Groningen, Groningen, Netherlands; ^4^ Department of Radiation Oncology, Shandong Cancer Hospital, Jinan, Shandong, China; ^5^ Department of Radiation Oncology, Olivia Newton-John Cancer Centre, Austin Health, Melbourne, VIC, Australia; ^6^ School of Medicine, Stanford University, Stanford, CA, United States; ^7^ Department of Radiation Oncology, The University of Texas MD Anderson Cancer Center, Houston, TX, United States

**Keywords:** radiomics, machine learning, cancer, biological behaviors, artificial intelligence, individual treatment

## Introduction

Cancer is a serious threat to human health worldwide, with a high mortality rate and increasing morbidity over the years (1). In general, patients with the same clinical stage have significant differences in survival and prognosis due to the high heterogeneity of tumor biological behavior. It is challenging to individualize treatment according to a uniform tumor stage treatment model. Radiomics is one of the indispensable tools for screening, diagnosis, treatment, and follow-up of multiple tumors. Recent data suggest that advanced post-treatment anatomic imaging with post-processing and registration capabilities can be used to characterize the likelihood and the location of potential failures to optimize treatment strategies and improve quality of life

With the wide application of artificial intelligence, the emerging radiomics technology, as a quantitative and high-throughput radiology method, has shown the ability to obtain quantitative texture information from existing medical image data from anatomical structures non-invasively, and has become the gold standard for pre-treatment staging and post-treatment tumor control evaluation (2). In addition, radiomics can further leverage existing “big data” analysis of images to provide hitherto unimaginable predictive power. It leverages powerful big data/machine learning techniques to refine its approach to massive data processing to identify clinically applicable, non-invasive methods to extract oncology outcomes and toxicity prevention data from large-scale data. This approach offers superior scalability, clinical applicability, cost-effectiveness, ease of implementation and an unmatched value proposition

## Papers included in this Research Topic

Radiomics is playing an increasing role in cancer diagnosis and treatment as well as biological research. We introduced this Research Topic to develop a comprehensive predictive model that provides standard recommendations for the multidisciplinary management of multiple cancer causes.The long-term goal is to reduce the mortality rate of cancer patients and improve their quality of life, thereby increasing cost efficiency. We are encouraged by the strong support of the research community for our Research Topic. We were very pleased to see many excellent works submitted to our research project, and we finally published 16 papers, including 13 original studies and 3 reviews, most of which were retrospective studies. The thesis covers brain glioma, nasopharyngeal carcinoma, breast cancer, small cell lung cancer, renal cell carcinoma, hepatocellular carcinoma, pancreatic cancer, rectal cancer and other tumors. The objectives of the study are diverse, including tumor status assessment, differential diagnosis, survival and recurrence assessment, and genomic feature prediction.

Accurate identification and evaluation using radiomics is helpful to develop appropriate treatment plans for patients and avoid unnecessary treatment measures such as surgery, postoperative radiotherapy and chemotherapy. For example, Wang et al. analyzing mammography (DM) images using radiomics, a radiomic line model was established to distinguish benign and malignant circular masses. Gao et al. used radiomics features based on enhanced CT images from the corticomedullary stage (CMP) and nephrography stage (NP) in combination with important clinical factors to distinguish between papillary renal cell carcinoma type 1 (pRCC) and pRCC type 2 tumors by multivariate logistic regression analysis before surgery. In addition, Lu et al. built a radiomic nomogram model based on CT images for patients with focal autoimmune pancreatitis and pancreatic ductal adenocarcinoma in accurate areas. The AUC of the training group and the test group were 0.87 and 0.83, respectively. As a non-invasive predictive tool, the model can improve the accuracy of diagnosis while reducing patient trauma and achieving optimal compliance. The classification and type of tumors are different, and the treatment methods are not consistent. For example, according to the multitask learning model developed by Huang, Y. et al. in combination with support vector machines to distinguish glioblastoma from isolated brain metastases, the mean AUC of the model in the training set and validation set was as high as 0.993 and 0.987. If the preoperative prognosis is primary glioblastoma, aggressive triple therapy, such as postoperative concurrent chemoradiotherapy, is required.

Image-based radiomics models can assist clinicians in treatment evaluation, including predicting the response of individual cancer patients to chemotherapy or immunotherapy, as well as monitoring recurrence and metastasis. These aspects are clearly reflected in the research papers accepted for this radiomics Research Topic: Jiang et al., Yang et al., Wang, Y. et al., Lin et al., and Huang, Y.-M. et al. used several radiomics based ML models and columns to predict patient outcomes, such as survival, mortality, efficacy, postoperative metastasis, and recurrence. Wang, Y. et al.‘s radiomics model based on GIST morphological features plays an important role in tumor risk stratification, and the AUC value of the model is 0.933.It can provide reference for clinical diagnosis and treatment plan, formulate the best treatment strategy for individuals according to the predicted results of the model, create customized treatment plan for patients, and improve the treatment effect and later quality of life.

## Conclusion

Radiomics has shown promising results in some areas of oncology, including tumor screening, detection, diagnosis, treatment, and prognosis prediction. The 16 studies collected under this study theme apply radiomics to construct comprehensive predictive models to provide optimal recommendations for multidisciplinary management of multiple tumors to address treatment options in clinical practice. Imaging has demonstrated the potential to improve the foresight and accuracy of the diagnosis and treatment of cancer patients. It is promising to applicate radiomics in clinical practice to improve the efficiency of clinicians, reduce the possibility of clinical decision-making errors, and reduce unnecessary procedures, interventions, and medical costs.

All the studies in the subject of this study are retrospective and have certain limitations. The data levels included in each study are not sufficient, and there is no unified standard reference for algorithms such as image source, lesion delineation and feature extraction. These studies are still complex for clinicians and difficult to be thoroughly accepted. More research may need to focus on image and data standardization between different institutions, data sharing, and prospective studies to increase generalization of results.

This Research Topic involves a number of studies and presents the application of radiomics in understanding tumor biological behavior and treatment response. We thank all reviewers and authors for their contributions to this Research Topic. We hope that this Research Topic will attract more attention in related fields.

## Author contributions

NX: Writing – original draft. ZP: Writing – original draft. WL: Writing – original draft. RF: Writing – original draft. YJ: Writing – review & editing. GZ: Writing – review & editing. XM: Writing – review & editing. SN: Writing – review & editing. LX: Writing – review & editing. ZL: Supervision, Writing – review & editing. NS: Supervision, Writing – review & editing. PY: Supervision, Writing – review & editing.
